# A MODEL FOR STUDENT PARTICIPATION IN COMMUNITY-ENGAGED RESEARCH FOR OLDER ADULTS: THE ENROLL PROGRAM

**DOI:** 10.1093/geroni/igac059.3063

**Published:** 2022-12-20

**Authors:** Matthew Peterson, Tina Newsham, Alicia Sellon, Madison Norris, Leanza Sidey, Christian Barnes, William Holland, Jodi Rich

**Affiliations:** University of North Carolina Wilmington, Wilmington, North Carolina, United States; UNC Wilmington, Wilmington, North Carolina, United States; University of North Carolina Wilmington, Wilmington, North Carolina, United States; University of North Carolina Wilmington, Wilmington, North Carolina, United States; University of North Carolina Wilmington, Wilmington, North Carolina, United States; University of North Carolina Wilmington, Wilmington, North Carolina, United States; University of North Carolina Wilmington, Wilmington, North Carolina, United States; New Hanover County Parks & Gardens, Wilmington, North Carolina, United States

## Abstract

The current academic climate has left naturally occurring faculty-led student research experiences and mentoring a challenge. This is coupled with an already existing dearth of future scientists interested in aging-related research. The overarching goal of the Enhancing Research for OnLine Learners (EnROLL) Program is to facilitate research collaboration among undergraduate minoritized students, graduate distance learners, health sciences faculty, and academic partners. The program includes a variety of strategic initiatives, one of which includes building five inter-institutional research cores: epidemiology, preclinical, allied health, applied learning, and community engagement. Here, we present a community-based participatory project led by students and researchers in the EnROLL community engagement core in partnership with local government. Researchers at UNCW were contacted by New Hanover County Parks & Gardens staff to assist in wellness planning for area older adults. Two surveys were developed: Nf103 community members and Nf8 staff members completed separate surveys. Community respondents were 60.8 +/- 13.7 years old, 72% female, and 80% Caucasian. Approximately 58% of community respondents were at least somewhat likely to participate in wellness programming at County locations. Based on preferred days, times, and types of classes of the respondents, a comprehensive weekly schedule of wellness classes was developed and provided to County staff for implementation under the guidance of a UNCW masters student. This community engagement project, and the EnROLL program, can serve as a model for student-engaged, aging-related research in the health sciences, particularly as gerontologists work to diversify the demographics of researchers who focus on aging.

